# Analysis of CRISPR/Cas Genetic Structure, Spacer Content and Molecular Epidemiology in Brazilian *Acinetobacter baumannii* Clinical Isolates

**DOI:** 10.3390/pathogens12060764

**Published:** 2023-05-26

**Authors:** Adrianne M. A. Silva, Ana C. O. Luz, Keyla V. M. Xavier, Maria P. S. Barros, Hirisleide B. Alves, Marcus V. A. Batista, Tereza C. Leal-Balbino

**Affiliations:** 1 Departamento de Microbiologia, Instituto Aggeu Magalhães, Fundação Oswaldo Cruz, Recife CEP 50740-465, Pernambuco, Brazil; adrianneasilva@gmail.com (A.M.A.S.); acarol.luz@gmail.com (A.C.O.L.); keylaxavier01@gmail.com (K.V.M.X.); hirisleidebezerra@gmail.com (H.B.A.); 2 Laboratório de Bioprocessos, Centro de Tecnologias Estratégicas do Nordeste, Recife CEP 50740-545, Pernambuco, Brazil; mariapalomabarros@gmail.com; 3 Laboratório de Genética Molecular e Biotecnologia, Centro de Ciências Biológicas e da Saúde-CCBS, Universidade Federal de Sergipe, Aracaju CEP 49060-108, Sergipe, Brazil; genetics.marcus@gmail.com

**Keywords:** spacer, phage, CRISPR/Cas, molecular epidemiology

## Abstract

CRISPR/Cas is a molecular mechanism to prevent predatory viruses from invading bacteria via the insertion of small viral sequences (spacers) in its repetitive locus. The nature of spacer incorporation and the viral origins of spacers provide an overview of the genetic evolution of bacteria, their natural viral predators, and the mechanisms that prokaryotes may use to protect themselves, or to acquire mobile genetic elements such as plasmids. Here, we report on the CRISPR/Cas genetic structure, its spacer content, and strain epidemiology through MLST and CRISPR typing in *Acinetobacter baumannii*, an opportunistic pathogen intimately related to hospital infections and antimicrobial resistance. Results show distinct genetic characteristics, such as polymorphisms specific to ancestor direct repeats, a well-defined degenerate repeat, and a conserved leader sequence, as well as showing most spacers as targeting bacteriophages, and several self-targeting spacers, directed at prophages. There was a particular relationship between CRISPR/Cas and CC113 in the study of Brazilian isolates, and CRISPR-related typing techniques are interesting for subtyping strains with the same MLST profile. We want to emphasize the significance of descriptive genetic research on CRISPR loci, and we argue that spacer or CRISPR typing are helpful for small-scale investigations, preferably in conjunction with other molecular typing techniques such as MLST.

## 1. Introduction

Due to the broad range of environmental niches prokaryotes can dwell in, they are frequently exposed to mobile genetic elements (MGEs), such as bacteriophages, plasmids, and transposons. Even though these interactions may be beneficial through the acquisition of new resistance and virulence-inducing genes, they may also lead to bacterial lysis promoted by phages [1]. Therefore, prokaryotes evolved different approaches to defend against viral infections and general exposure to MGEs. The CRISPR/Cas system is one of these mechanisms, and it acts as an adaptive immune system, capable of producing and storing genetic memories of previous encounters of the cell lineage with MGEs [2,3].

This system consists of two key elements: the CRISPR (clustered regularly interspaced short palindromic repeats) locus, and the Cas (CRISPR-associated) proteins. Direct repeats (DRs) and spacers are the elements that comprise the CRISPR locus and are responsible for the characteristics of this system, since previous encounters with MGEs generate new spacers and, when transcribed, these spacers function as RNA guides for the degradation of such MGEs. Cas proteins, the products of *cas* genes located adjacent to CRISPR loci, have broadly distinct functions, and act together with CRISPR RNAs (crRNAs) to prevent new infections from previously encountered MGEs [4,5,6].

Phages developed defense mechanisms of their own, including anti-CRISPR proteins that can disable the CRISPR/Cas system, in a process akin to an arms race. Anti-CRISPR elements and microbial diversity prompt the evolution of this system, making this immunity mechanism highly variable and diverse [7,8,9]. Given its ability to avoid the host’s defenses, phages can integrate their genomes as prophages, until environmental factors or bacterium signaling induces its expression. Prophage regions can remain dormant and be passed on to offspring, inserted within the bacterial chromosome. However, with subsequent cell divisions, some of the prophage genes may accumulate point mutations and the phage regions become non-inducible. These non-inducible or cryptic phages, if harboring antimicrobial determinants or virulence factors, act as a gene reservoir for the bacteria [10].

Phages can integrate their genomes as prophages because they can bypass the host’s defenses until environmental circumstances or bacterial signaling trigger its production. Prophage sections can be inserted into the bacterial chromosome, where they can lay dormant and be passed on to progeny. However, some prophage genes may develop point mutations with subsequent cell divisions, making the phage regions non-inducible. If these cryptic or non-inducible phages contain virulence or antibacterial characteristics, they serve as a gene reservoir for the bacterium.

*Acinetobacter baumannii* is a Gram-negative opportunistic coccobacillus, able to resist environment variations, and thus able to persist on abiotic surfaces for extended periods of time. With these traits, it successfully colonizes infirmaries and intensive care units (ICUs) and may cause important hospital-related infections. Furthermore, *A. baumannii*’s resistance to disinfection, and increasing multidrug resistance frequencies, may also lead hospital equipment and infected patients to become reservoirs that promote this species’ endurance and spreading [11,12]. In view of the ever-growing mortality rates of *A. baumannii*-related infections, treatment difficulties, transmissible capacities, and resistance to antibiotics in the carbapenems class, the World Health Organization (WHO) classified this species, along with other concerning pathogens, in the Critical Priority tier for new drug development [13].

Previous authors have reported the presence of CRISPR/Cas systems in *A. baumannii* isolates, reporting system types I-F1 and I-F2 [14,15]. However, few studies have focused on *A. baumannii*’s CRISPR/Cas system, its genetic sequence, structure, associated elements, spacer content or activity [14,16,17,18,19]. Only a handful of works have used CRISPR/Cas, and mostly for strain typing [20,21,22]. One work alone [23] has provided information about the CRISPR/Cas system scenario in Brazilian strains; however, they did not analyze the system’s structure, spacer content, or phage association. Our goal was instead to conduct a CRISPR/Cas-focused research in Brazilian clinical specimens of *A. baumannii*.

Here, we aimed to perform the first focused analysis on the CRISPR/Cas systems of Brazilian clinical isolates of *A. baumannii*. It was our intention to help future researchers by establishing grounds for further descriptive structural CRISPR/Cas investigations in this bacterial species by describing its sequence, genetic characteristics, spacer incorporation patterns, simultaneous phage occurrence, and anti-CRISPR content.

## 2. Results

### 2.1. CRISPR/Cas Genetic Structure in Brazilian A. baumannii Isolates

In total, 14 of the 47 isolates were CRISPR/Cas-positive genomes as they each had a single CRISPR locus with nearby *cas* genes, thus being classified as CRISPR/Cas subtype I-F. These strains were also confirmed as CRISPR/Cas-positive by CRISPRone, an online prediction tool with an integrated pipeline for checking false positives.

The CRISPR loci length ranged between 1.889 and 6.512 bp (base pair). The conserved elements from CRISPR, such as DRs and DG (degenerate repeat), presented the same length among Brazilian isolates, and DR sequences were identical to the control strain AYE, while the DG sequence differed from this strain. The first DR sequence after DG was counted as the first DR in the locus since DG denotes the start of a CRISPR locus and is situated on the opposite side from the leader sequence, where new spacers are incorporated; as a result, the spacer between DG and the first DR was the first spacer.

All isolates, except for Acb_8, carried identical point mutations in their first three DRs. Acb_8, however, presented divergence in its second and last DRs (Supplementary Materials S1 and S2).

All 14 CRISPR/Cas-positive genomes were associated with *cas1*, *cas3*, *csy1*, *csy2*, *csy3*, and *cas6f*, and thus we were able to assign them as type I, subtype F1, according to the most recent classification. The gene sequences were identical in most isolates and the control strain AYE, but Acb_41 had point mutations in its *cas1* and *cas3* genes (Supplementary Material S3). 

**Figure 1:**
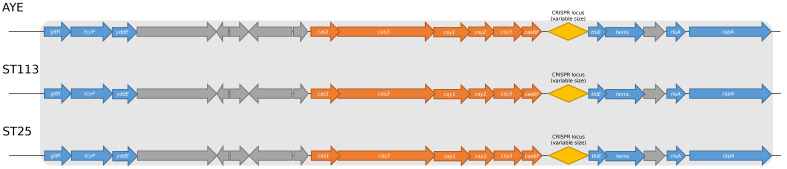
Comparison between the genetic background of strain AYE, used here as a reference for CRISPR/Cas systems, strains Acb_8 representing ST113, and strain Acb_41 representing ST25. Blue arrows: identified genes surrounding the CRISPR/Cas system; gray arrows: hypothetical protein genes; orange arrows: *cas* genes; yellow diamond shape: CRISPR locus; light gray rectangle: surrounding identical genes.

Multilocus sequence typing, previously performed by [24], identified seven STs (sequence types) among the initial 46 Brazilian isolates. However, we found that all CRISPR/Cas-positive isolates belonged to ST113, except for Acb_41 (ST25). Both STs were from the same clonal complex (CC), called CC113, according to the Pasteur MLST scheme. We compared the CRISPR/Cas genetic background, which means the proteins surrounding this system in the genomes studied, of the Brazilian strains studied with the AYE genetic structure, and all of these strains presented the same pattern (Figure 1).

No comparable sequences for anti-CRISPR genes were identified. Two different search methods confirmed that these Brazilian isolates do not carry known anti-CRISPR elements. A comparison between positive isolates and AYE allowed us to identify the possible leader sequence, with the characteristics expected, e.g., the placement in the genome (upstream CRISPR locus), size (147 bp), and low GC content (25.9%) (Supplementary Material S2).

### 2.2. Spacer Analysis

The pool of spacers among the 14 CRISPR/Cas-positive isolates contained 152 spacers, ranging from 32 to 34 bp (Supplementary Material S4). Some spacers presented polymorphisms, such as s22 in Acb_41, when compared with this same spacer in Acb_5 and Acb_29, but if these differences were fewer than four point mutations (SNP), we considered these spacers as the same, to avoid misidentification and possible sequencing errors. The exception was s92, that presented two variants differing in two bases each, but because these polymorphisms were in various positions in CRISPR loci, they were indicative of mutations.

A total of 38 new spacers (38/152, 25%) had already been submitted to the NCBI database in CRISPR sequences of deposited genomes, 20 (20/152, 13.2%) were submitted to CRISPRdb, and 10 (10/152, 6.5%) were simultaneously found in both these databases. Thus, we considered the 84 (84/152, 55.3%) remaining spacers found in the Brazilian isolates as new spacers, meaning they were never found and/or restricted to Brazilian genomes.

Spacer sequences showed similarity not only to MGEs but also to the proteins from the bacteria’s own metabolism (Supplementary Material S4). We were also able to find spacer correspondence for uncharacterized proteins deposited in the databases, and spacers with no correspondence to any database.

Among spacer sequences, 60 (60/152, 39.5%) were related to phages, 12 (12/152, 7.89%) were related to plasmids, and 26 (26/152, 17.1%) were related to both phages and plasmids. A total of 2 (2/152, 1.3%) were similar to the VirB4 protein, a component of the Type IV Secretion System (T4SS) and were consequently characterized as self-targeting spacers, and 50 (50/152, 76%) spacer sequences showed no significant relation to any known sequence.

All phage-related spacers showed similarity to at least one previously known phage, with s33 being the exception as it corresponded to a phage not yet described. Several spacers corresponded to phages unrelated to the genus *Acinetobacter*, such as s82 with phage NATL1A-7, specific to cyanobacteria, and s74, with phage CrAss-like virus, specific to the genus *Bacteroides*, for example. Eight spacers corresponded to archaeal genes.

Most spacers were related to more than one phage. For example, s109 shared similarities with 143 distinct phages infecting 108 bacterial genera, such as *Pseudomonas* spp., *Salmonella* spp., *Escherichia* spp., *Nissabacter* spp., and *Cronobacter* spp.

### 2.3. Prophage Identification

The prophages were identified as intact, questionable, or incomplete by PHASTER. All CRISPR/Cas-positive isolates carried at least one prophage-related region, except for Acb_29 and Acb_41, which we found either possessing no intact regions (Acb_29) or only incomplete regions (Acb_41) (Table 1). We identified 8 *Acinetobacter* spp. phages, of which 5 (5/8, 62.5%) were associated with questionable regions, and the remaining 3 (3/8, 37.5%) were intact. 

The intact phages were estimated as complete functional phages. Questionable and incomplete phages that did not contain sufficient prophage genes were considered as unfunctional phages (cryptic) [25,26]. 

Comparing these data with spacer origins, we found ten genomes carrying prophages and spacers capable of targeting these prophages (Supplementary Material S2). For example, prophage vB_AbaS_TRS1 was found in Acb_5, Acb_24, and Acb_29, even though all these isolates carried spacers s75 and s79, likely to have this phage as its origin.

### 2.4. CRISPR Typing

We compared the CRISPR types obtained to CRISPR sequence types (CST) established previously for *A. baumannii* [14], and 104 spacers were identical. All isolates showed similarity with CST14, except for Acb_41, which shared similarities with CSTs 23 and 24. Isolates Acb_1, Acb_8, Acb_21, Acb_24, Acb_33, Acb_35, Acb_36 and Acb_38 shared 19 spacers with CST14; Acb_5 and Acb_29 shared 64 spacers with CST14; Acb_44 and Acb_47 shared 48 spacers with CST14; and Acb_4 shared 52 spacers with CST14. The Acb_41 isolate shared 40 spacers with both CST23 and CST24. 

However, none of them carried the necessary spacers to properly fit a CST pattern. We thus propose grouping isolates Acb_1, Acb_8, Acb_21, Acb_24, Acb_33, Acb_35, Acb_36, and Acb_38 into a new CST, CST76; isolates Acb_5 and Acb_29 in CST77; Acb_4, Acb_44 and Acb_47 in CST78; and isolate Acb_41 in CST79. A full depiction of newly assigned CSTs can be found in Supplementary Material S4.

**Figure 2:**
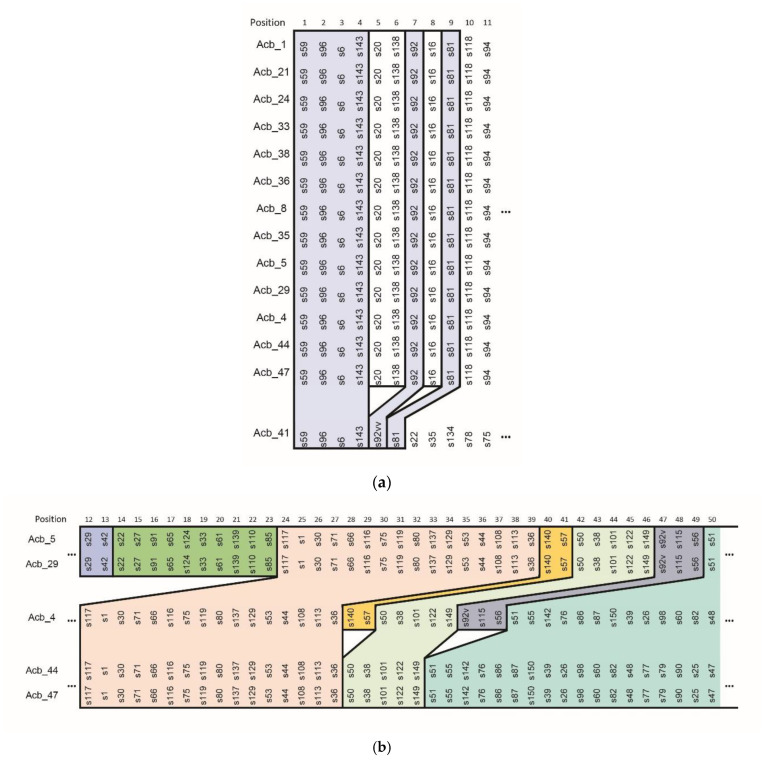
Spacer rearrangements in *A. baumannii* isolates. Spacers are depicted by their assigned name (the letter “s” followed by a number), and colors are representative of sets of spacers incorporated in the same order, showing rearrangements that occurred between lineages. (**a**) First 11 spacers from all CRISPR/Cas-positive Brazilian isolates, and (**b**) isolates Acb_4, Acb_5, Acb_29, Acb_44, and Acb_47 between positions 12 and 50. Full CRISPR portrayal can be found in Supplementary Material S5.

CRISPR typing, as described by previous authors [14,27,28] is defined by the comparison of spacer sequences and their incorporation order (Supplementary Material S5), and by comparing the order of spacer incorporation in Brazilian isolates, rearrangements within CRISPR loci (Figure 2) were observed.

Acb_41 shares only the first four spacers with the other isolates. Two additional spacers, located in positions five and six, are shared, and their positioning suggests that a rearrangement occurred (Figure 2a). The other isolates, which showed more genetic similarity between them, are genetically more distant from Acb_41, according to CRISPR-typing alone.

Strains Acb_5 and Acb_29 carry an exclusive subset of spacers, from positions 14 to 23. In total, 2 other subsets are shared with Acb_4 only, from positions 40 and 41, and 47 to 51. The remaining spacers are the same as strains Acb_44 and Acb_47, in both sequence and incorporation order (Figure 2b). As a result of this, we concluded that these two subgroups are more closely related.

### 2.5. Phylogenetic and Epidemiologic Approaches

**Figure 3:**
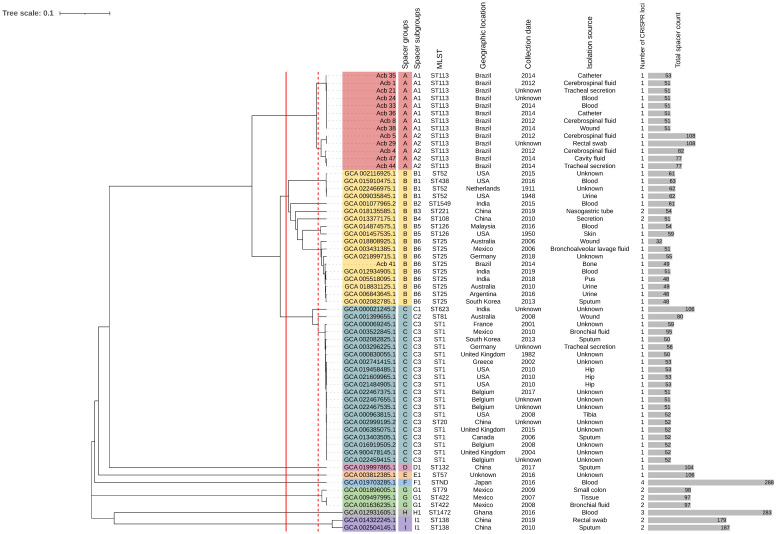
Dendrogram showing the relationship between *A. baumannii* isolates, according to their spacer content. Nine spacer groups (from A to I) were defined by the cut-off at the red continuous line; and seventeen subgroups were defined by the cut-off at the red dashed line. Spacer groups are designated by distinct colors, and spacer subgroups are identifiable by colors text labels. Strains epidemiology and genetic information, such as MLST, geographical location, collection date, isolation source, number of CRISPR loci and the sum of their spacer count, are listed in the right side of the dendrogram. STND stands for non-determined ST.

We took two approaches in this study for CRISPR-based typing techniques: CRISPR-typing, which has been previously explored, and the analysis of spacer sequences disregarding their positioning in CRISPR loci and combining them in artificial sequences, from now on named spacer-typing. With the goal of expanding our analysis, since the CRISPR/Cas system was restricted to two STs among the Brazilian genomes, we added 47 publicly available *A. baumannii* genomes, which were CRISPR/Cas-positive and collected from clinical onsets to spacer-typing, and strains were divided in spacer groups and subgroups according to their spacer content similarity (Figure 3). These additional strains’ information and epidemiology aspects are available in Supplementary Material S6, and their genetic background, as previously performed for the Brazilian strains and AYE, are available in Supplementary Material S7.

The isolates were grouped into nine primary spacer groups, with spacer subgroups being able to further distinguish between them. Group A comprised all Brazilian isolates belonging to ST113 and divided these strains into two subgroups (A1 and A2). Eight Brazilian strains made up spacer subgroup A1, making it the A subgroup with the most strains. The strains in this subgroup varied in their collection dates and isolation sources. Except for Acb_35, which had two extra spacers at the end of its CRISPR locus, every other strain in this subgroup had the same number of spacers. According to their positioning, these spacers were the last to be acquired, which does not imply that this strain belongs to a new subgroup yet, but that a new subgroup might emerge from it in the future.

We also compared spacer typing with other CRISPR/Cas-related typing methods, using *cas1* sequences. Unexpectedly, *cas1* sequences from CRISPR/Cas subtypes I-F1 and I-F2 did not divide themselves in different groups, even though this technique was also able to discern between strains, but less accurately than spacer-typing (Supplementary Material S8).

**Figure 4:**
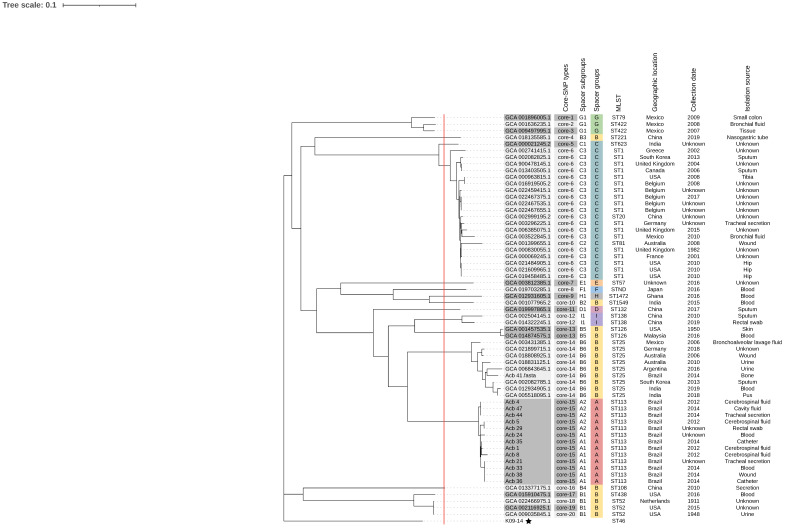
Dendrogram based on SNPs in the core genome of *A. baumannii*. CRISPR subgroups assigned by spacer-typing for each strain are listed in the right side of the dendrogram. Twenty core groups were defined based on the cutoff at the red continuous line and are depicted by different shades of gray. Correspondent spacer groups and subgroups previously determined are found in the right side of the dendrogram, as well as other epidemiologic information. Black star indicates the reference genome according to NCBI, and used as reference for core SNPs (*A. baumannii strain* K09-14, CRISPR/Cas-negative, assembly GCF_008632635.1).

To identify if the spacer groups and subgroups established would maintain themselves with a broader, genome-based typing technique, we performed the analysis of single nucleotide polymorphisms (SNPs) in the core-genome (core-SNP). Since the full core-genome was examined, the resulting dendrogram has a stronger capacity to discriminate closely related strains, as would be predicted, but the overall outcome of spacer-typing is easily comparable to the core-SNP dendrogram, as seen in Figure 4.

Core-SNP dendrogram divided the strains into 20 different groups, here called core-SNP groups in order to compare them with the spacer-groups determined by the spacer-typing technique. Aside from two spacer subgroups (B1 and G1), which were further subdivided in the core-subgroups, all other assigned core-subgroups were directly corresponded to the assigned spacer-subgroups, confirming the high discriminatory power the analysis of spacers from CRISPR loci can have when using this loci for molecular typing.

Spacer-typing and core-SNP based dendrograms confirmed the hypothesis made with only CRISPR-typing, such as the relationship of Acb_4, Acb_44, and Acb_47 (A2) with Acb_5 and Acb_29 (A2). The distance between Acb_41 and the other Brazilian strains is also present in these dendrograms, as Acb_41 (B6) is grouped with other ST25 strains in spacer group B. According to their ST profiles, STs 113 and 25 are parts of the same clonal complex, which can be seen by their closeness both in spacer-typing dendrogram, as well as in core-SNPs. 

## 3. Discussion

It is well established that multidrug resistance makes it possible for strain maintenance and circulation in hospital settings [29]. CRISPR/Cas systems are meant to protect bacteria from invasion by MGEs, and it is reasonable to believe that the action of this system may interfere with the acquisition of new virulence from resistance genes carried by plasmids or even by phages [30]. Since most isolates lack CRISPR/Cas (33/47, 70.2%), this could suggest that it is evolutionary disadvantageous for *A. baumannii* to preserve CRISPR/Cas systems, with natural selection favoring MGE acquisition and lower metabolic costs [31].

Traditional approaches for locating CRISPR loci rely heavily on looking for repetitive genetic regions separated by variable sequences. However, regions with such genetic structure can also occur in prokaryotic proteins and thus lead to false positive results [32]. In our research, four isolates appeared to have CRISPR loci even though there were no *cas* genes associated. Both softwares, CRISPRCasFinder and CRISPRone, confirmed the absence of the CRISPR/Cas systems in these isolates, along with the high similarity between their supposed spacers (87.5% similarity) [33].

The origin of some spacer sequences could not be determined, even if they were present in publicly available genomes or were previously submitted to CRISPRdb. It is common to find spacers with unknown origins, even in other species such as *Pseudomonas aeruginosa* [34] and *Yersinia pestis* [35]. These findings are probably the result of the overwhelming number of unidentified phages that have never been sequenced and the huge percentage of hypothetical and uncharacterized proteins in databases. Among phage-related spacers, we identified phages that may target more than one bacterial genus. This could occur based on horizontal gene transfer because CRISPR loci can be shared in this manner [31,36].

In seven isolates, we found prophages alongside spacers targeting these viral sequences. Phages Bphi_B1251 in isolates Acb_5 and Acb_21, Psymv2 in Acb_24 and Acb_44, and phiCTX in Acb_47, all correspond to intact regions and, thus, should be capable of induction. Bacteria may be keeping these prophages dormant through CRISPR/Cas action, with spacers acting as a guide not to self-target the genome but to control viral gene expression and prevent these prophages from initiating a lytic cycle [33,37]. The gene content in those regions, however, needs to be further analyzed to identify which ones can enter the lytic cycle and propagate.

Even though the primary function of CRISPR/Cas is to stop MGEs from replicating inside the bacterial cell, finding self-targeting spacers in viable cells is not rare, and there are CRISPR/Cas systems that seem more prone to acquiring them, such as subtype I-F [33]. Three spacers (s4, s144, and s145) are likely to target the protein Vir4B from T4SS, and six spacers (s15, s58, s64, s111, s123, and s126) are supposed to target uncharacterized proteins of *A. baumannii*. Since anti-CRISPR genes were absent from all CRISPR/Cas-positive isolates, cell viability may be related to other mechanisms of autoimmunity evasion.

Since spacer acquisition is organized in a chronological manner, with the newer spacers always incorporated in the leader sequence end of CRISPR loci, unrelated isolates, cultured in different settings and/or environments, are highly unlikely to carry the same set of spacers in the same order of incorporation. This very nature of spacer acquisition in CRISPR loci makes it an interesting tool for molecular typing of strains [38]. Even though rearrangements can occur [39] and appear to be quite frequent in *A. baumannii*, as our results indicate, strains with similar sets of spacers are more likely to be genetically related, as we observed in the spacer-typing dendrogram and confirmed with the core-SNP tree. The CRISPR/Cas control strain *A. baumannii* AYE has a ST1 profile (CC1), and even though it is more distantly placed in the phylogenetic tree previously elaborated [23], the leader sequences from both CCs were identical, and two spacers were shared, suggesting some level of conservation between these CCs.

In our initial set of strains, CRISPR/Cas appeared exclusively in CC113 strains (STs 113 and 25). Some strains had the same ST as previously known CRISPR/Cas positive lineages, such as ST1, but resulted as negative for the presence of this system [14]. The presence of CRISPR/Cas has already been reported for CC113/25 strains [13], but its presence exclusively in strains related to this CC in the Brazilian genomes here studied was not expected. Multidrug-resistant (MDR) strains belonging to ST 25 have already been found in Brazilian hospitals, and the CC113/25 is considered hyperendemic in Brazil [40,41]. Sahl et al. [42] reported that ST 25 isolates are highly diverse in their virulence phenotypes [38], which emphasizes the importance for new approaches to identify the evolutionary processes of ST 25 strains of *A. baummannii* [42]. 

Strains with ST 113 (group A) presented two different ramifications that, by observing the CRISPR-typing, we can establish is the result of the first eleven spacers that are shared between these subgroups. According to Karah in 2015, internal remodeling events, such as spacer deletions, duplications, and inserts, can occur in CRISPR loci and make up for different genotypes [14]. We can then infer that the CRISPR locus of Acb_4 is a product of the rearrangement of spacers from A3, finally culminating in the A2 spacer content.

Previous works reported the efficiency of CRISPR typing methods in other bacterial species, suggesting microevolutionary processes and a broader dissemination aspect in genetically similar or geographically close strains [27,28,35,43,44]. In *A. baumannii*, the analysis of spacer sequences appears to be an interesting method for subgrouping strains within the same ST profile. Since the spacer-typing dendrogram shows a high correlation between spacer groups and MLST and can further subgroup strains, but also has lower discriminatory power than core-SNP, it might be a useful subtyping method for local, short-term investigations of outbreaks and/or dispersion. According to Rafei et al. [45], other typing techniques to achieve these kinds of investigations are PFGE, MLVA, and RAPD. Spacer-typing may be most helpful in outbreak scenarios if used in conjunction with one of those techniques because not all *A. baumannii* isolates include the CRISPR/Cas system, but further work is recommended to support this hypothesis.

Another noteworthy argument is that spacer typing can be achieved with just one amplification because all its data are in a single genomic locus. This could enable quicker and cheaper further investigation.

Our results establish the typical sequences for CRISPR/Cas-related features in Brazilian strains of *A. baumannii*, that may be helpful for further structural genetic studies of this species. Our findings also indicate that CRISPR/Cas may also be regulating the expression of prophage products, and that typing and subtyping with CRISPR- and spacer-typing methods may be useful in studying *A. baumannii* evolution, and for distribution in small-scale research. To expand any epidemiological and phylogenetic analysis, spacer-typing, paired with other typing techniques, can become a robust method to assess *A. baumannii* clinical isolates.

## 4. Materials and Methods

### 4.1. Brazilian Bacterial Strains

This study initially included 47 *A. baumannii* clinical isolates collected and identified at the species level, provided by 5 public hospitals in Recife, State of Pernambuco, Northeast Brazil. We had no contact with patients nor patient information. These isolates were stored in glycerol-BHI at −80 °C at the Department of Microbiology, Aggeu Magalhães Institute. All isolates had been previously sequenced and made publicly available at European Nucleotide Archive (ENA) accession number PRJEB12754 [24].

For comparisons of CRISPR loci and *cas* genes sequences and features, we chose strain *A. baumannii* AYE (GenBank accession number GCA_000069245.1), usually considered as a model for CRISPR/Cas studies [14].

### 4.2. CRISPR/Cas Analysis

CRISPR loci investigation was performed with CRISPRFinder [46] and confirmed with CRISPRone [32]. Degenerate repeats (DG) were found and defined manually, since the tools cannot recognize these sequences accurately.

BLASTn in NCBI and Uniprot were used to confirm and compare previously annotated *cas* genes sequences. We considered only results whose e-value was lower or equal to 1.0 [47]. According to the most recent CRISPR/Cas classification [9], CRISPR/Cas typing was assigned. Genetic architecture was observed with Artemis [48]. Further analyses were performed only with CRISPR/Cas-positive genomes.

### 4.3. Anti-CRISPR Investigation

Anti-CRISPR sequences specific to subtype I-F were obtained from AcrHub [49] and investigated in genomes through alignment using BLASTx (translated BLAST). To confirm the results, we also used AcrFinder [50].

### 4.4. Leader Sequence

We checked the leader sequences manually, through comparison with the reference strain AYE, considering its pre-established characteristics (low GC content and positioning within the CRISPR/Cas system) [25,51].

### 4.5. Spacer Analysis

Spacers were named using a letter (s) and a number (1–150), according to the alphabetic order of its bp contents. Sequences whose differences were up to four bp were considered the same spacer to avoid sequencing errors, while for differences between four and seven bp, they were considered as a variant of the original spacer. The spacer named s92 and its variants were exceptions to this rule because the distribution in the CRISPR loci of this spacer and its variants was indicative of spacer mutation. Spacer order was numbered from DG, thus the first (or oldest) spacer from a CRISPR locus is the one closest to DG and the furthest from the leader sequence. We compared all sequences to CRISPRdb and GenBank using BLASTn to determine if similar spacers had already been found. New spacers were the ones not found in these databases.

CRISPRTarget [51] was applied to determine possible spacer origins, using default configurations. Results of BLASTn research were compared against GenBank and Uniprot databases were developed with the same purpose, and only the highest scores and lowest e-values (<1.0) were considered.

### 4.6. Bacteriophage Investigation

PHASTER online tool was used to identify prophage regions [25]. PHASTER uses methods to categorize the genome’s prophage regions, and it classifies these regions as intact, questionable, or incomplete. 

Results are classified as incomplete if these regions receive a score of less than 70, questionable if they receive a score between 70 and 90, and intact if they receive a score of more than 90. In this study, the presence of a phage was considered if: (1) phage completeness was shown to be intact, or (2) phage completeness was shown to be questionable, but BLAST results needed to return at least 50% coverage. The sequences for intact and questionable prophage regions were compared to our CRISPR spacers library using BLASTn provided by NCBI, to identify possible CRISPR/Cas system control upon those phages.

### 4.7. Phylogeny and Epidemiology Analysis

Alongside the Brazilian isolates explored in this study, we added 47 publicly available *A. baumannii* CRISPR/Cas-positive genomes when performing MLST, other CRISPR/Cas system-related typings and core-genome typing (Supplementary Material S6). These strains were obtained from the CRISPRCasFinder online database of strains (available at https://crisprcas.i2bc.paris-saclay.fr/, accessed on 2 May 2023). Since *A. baumannii*’s reference genome, strain Abaum K09-14 (assembly GCF_008632635.1), is negative for the CRISPR/Cas system, it was used as a reference in core genome analysis but is absent in the other typing methods.

MLST was determined through typing in pubMLST (available at https://pubmlst.org/organisms/acinetobacter-baumannii, accessed on 2 May 2023), using the Pasteur scheme as a default [52]. The MLST of Brazilian isolates, however, has been previously determined [24].

A comparison of CRISPR loci between strains, here referred to as spacer-typing, was made by converting the spacer content of CRISPR loci to a binary matrix according to spacer presence or absence and disregarding their position in CRISPR loci. All spacers were obtained through CRISPRCasFinder [53], from CRISPR loci with evidence level of four. Some CRISPR loci with evidence level of two, but with more than three spacers and a similar DR sequence to level four loci, were also included. These spacers were all converted to the positive potential orientation, if needed, based on their DR sequence and orientation inference from the CRISPRCasFinder tool. Binary matrix was obtained by constructing a genetic artificial sequence for each strain and assigning each position of this artificial sequence to a single spacer. Presence would mean an A in that specific position, and absence would mean a T. Thus, we obtained artificial sequences with each position of these sequences referencing a different spacer, that were then converted to FASTA format. Since positions are important in this case, we built an UPGMA tree using MEGA11 [54] without prior alignment, thus preserving sequence integrity, applying default configurations, and a bootstrap of 10,000.

CRISPR loci were also compared to previously established spacer dictionaries for *A. baumannii* [14] to determine a similar CRISPR Sequence Type (CST).

Additional CRISPR/Cas-related dendrograms were obtained using the *cas1* genes. Sequences from all isolates (disregarding if subtypes I-F1 or I-F2) were submitted for alignment, phylogeny, and tree rendering with phylogeny.fr (available at http://www.phylogeny.fr/, accessed on 2 May 2023) using default parameters [55]. To attempt to identify if spacer-typing, as performed in this study, was effective as a typing method for *A. baumannii* and could be correlated to genomic-based typing method, a dendrogram based on the alignment of the core genome was obtained with Parsnp [56]. All dendrograms were edited with Itol [57].

Genetic backgrounds, as in the analysis of surrounding genes, for CRISPR/Cas-positive strains were obtained by isolating a fraction of 40,000 pb of their genome, with *cas1* gene in the center. We submitted these targeted sequences to Prokka [58] annotation in order to standardize their gene names. Then, we compared the genes surrounding the CRISPR/Cas systems, keeping only one representative per ST, unless another strain within that ST showed mutations or a different genetic background.

## 5. Conclusions

Since all Brazilian isolates carried the same polymorphisms in the ancestor DRs, the same signature DG, and an identical leader sequence, the sequences here described could be helpful for the further description of CRISPR/Cas genetic structure in this species and be used to ensure full coverage of CRISPR loci. Spacer analysis indicates the importance of phages in *A. baumannii* evolution and emphasizes the need for further knowledge on methods for evading CRISPR/Cas immunity function. We can conclude that in *A. baumannii*, spacer-typing, together with MLST, may be an interesting subtyping method for small-scale phylogenetic and epidemiologic studies of clinical *A. baumannii* isolates with identical ST profiles. However, in larger studies, we would recommend spacer-typing in addition to other typing techniques for accurately discerning between strains.

## Figures and Tables

**Table 1:** Prophages in *A. baumannii* CRISPR/Cas-positive isolates. Regions types were marked with colors: intact (green), questionable (grey).

Isolates	Size in Kb	Prophage
Acb_1	21.1	PHAGE_Haemop_SuMu_NC_019455
Acb_4	17.9	PHAGE_Acinet_Bphi_B1251_NC_019541
	39.4	PHAGE_Haemop_SuMu_NC_019455
	34.6	PHAGE_Haemop_SuMu_NC_019455
Acb_5	43.4	PHAGE_Acinet_Bphi_B1251_NC_019541
	31.3	PHAGE_Pseudo_B3_NC_006548
	51.4	PHAGE_Acinet_vB_AbaS_TRS1_NC_031098
	38.2	PHAGE_Acinet_vB_AbaS_TRS1_NC_031098
Acb_8	21.1	PHAGE_Mannhe_vB_MhM_3927AP2_NC_028766
Acb_21	39.3	PHAGE_Mannhe_vB_MhM_3927AP2_NC_028766
	37.4	PHAGE_Pelagi_HTVC010P_NC_020481
	42.6	PHAGE_Acinet_Bphi_B1251_NC_019541
Acb_24	38	PHAGE_Acinet_vB_AbaS_TRS1_NC_031098
	21.1	PHAGE_Haemop_SuMu_NC_019455
	42.8	PHAGE_Acinet_Bphi_B1251_NC_019541
	66.9	PHAGE_Psychr_Psymv2_NC_023734
Acb_29	17.9	PHAGE_Haemop_SuMu_NC_019455
	43.6	PHAGE_Acinet_vB_AbaS_TRS1_NC_031098
	10.5	PHAGE_Psychr_Psymv2_NC_023734
	23.4	PHAGE_Mannhe_vB_MhM_3927AP2_NC_028766
Acb_33	18.1	PHAGE_Psychr_Psymv2_NC_023734
	28.9	PHAGE_Mannhe_vB_MhM_3927AP2_NC_028766
Acb_35	16.6	PHAGE_Psychr_Psymv2_NC_023734
	39.8	PHAGE_Mannhe_vB_MhM_3927AP2_NC_028766
Acb_36	21.9	PHAGE_Mannhe_vB_MhM_3927AP2_NC_028766
Acb_38	24.3	PHAGE_Haemop_SuMu_NC_019455
	16.6	PHAGE_Psychr_Psymv2_NC_023734
Acb_41	-	-
Acb_44	36	PHAGE_Psychr_Psymv2_NC_023734
	38.5	PHAGE_Pseudo_Dobby_NC_048109
	36.9	PHAGE_Haemop_SuMu_NC_019455
Acb_47	38.2	PHAGE_Escher_ECP1_NC_049926
	13	PHAGE_Pseudo_phiCTX_NC_003278
	41.6	PHAGE_Mannhe_vB_MhM_3927AP2_NC_028766
	7	PHAGE_Mannhe_vB_MhM_3927AP2_NC_028766

## Data Availability

All isolates are publicly available at the European Nucleotide Archive (ENA) accession number PRJEB12754. Further data are available in this article or its Supplementary Material.

## Supplementary Materials

The following supporting information can be downloaded at: https://www.mdpi.com/article/10.3390/pathogens12060764/s1. Supplementary Material S1: Graphic scheme of CRISPR locus from *A. baumannii* isolates, based on isolate Acb_1 (colors represent the elements inside a CRISPR locus: yellow—DR; Green—DG; Red—mutations in comparison with the standard DR; White—spacers). Supplementary Material S2: comparison between DR, DG, and leader sequences. Supplementary Material S3: *cas1* and *cas3* alignment between isolate Acb_41 and CRISPR/Cas reference strain AYE. Supplementary Material S4: dictionary of spacers, spacer origins in Brazilian *A. baumannii* genomes and new CST patterns. Supplementary Material S5: spacer order of incorporation for each Brazilian isolate, denoting rearrangements. Supplementary Material S6: availability and epidemiological information on the genomes used in phylogenetic analysis and Brazilian genomes. Supplementary Material S7: genetic background of CRISPR/Cas systems I-F1 and I-F2 from genomes used in phylogenetic analysis and Brazilian genomes. Colors represent genes: dark red—transposases of any kind; pink—integrases of any kind; yellow—*cas1*; gray—*cas3*; salmon—*csy1*; dark blue—*csy2*; green—*csy3*; light blue—*cas6f/csy4*; purple—CRISPR regions. There is one strain representing the ST, unless another strain in the same ST showed differences or mutations. We aligned strains by the *cas1* position. Supplementary Material S8: *cas1* dendrogram.
