# Supplementary material for: Analysis of CRISPR/Cas Genetic Structure, Spacer Content and Molecular Epidemiology in Brazilian Acinetobacter baumannii Clinical Isolates
Source: Pathogens. 2023 May 26;12(6):764. doi: 10.3390/pathogens12060764 (PMC10302819; doi:10.3390/pathogens12060764)
Supplement: Supplementary file 1 [file pathogens-12-00764-s001.zip › Supplementary material S2.pdf]

Conserved elements from CRISPR loci in *A. baumannii* Brazilian isolates and control strain *A. baumannii* AYE. Gray marks signal point mutations and insertions when comparing with a standard DR sequence.

CRISPR element	Isolates	Sequence 5′-3′	Size in pb	Placement in CRISPR locus
DR	All	GTTCATGGCGGCATACGCCATTTAGAAA	28	DRs
DG	All	GTTCATGACGGTATACGTCACTTAAGCG[1]	28	Opposing side of leader sequence
DR	Acb_8	AGTTCATGGCGGCACACGTCATTTAGAAA	29	Second DR
DR	Acb_8	GTTCTTGGCGGCATACGCCATTTAGAAA	28	Last DR
DR	All except Acb_8	GTTCATGGCGGCACACGTCATTTAGAAA	28	Second DR
DR	All	ATTCATGACGGCATACGTCATTTAGAAA	28	First DR
DR	All	GTTCATGTCGGCATACGCCATTTAGAAA	28	Third DR

Leader sequence observed in *A. baumannii* isolates and control strain.

Sequence 5′-3′
GTCCCAGAGTTTTGACCCAATATTTTTTCTATTCTTTAACAGCTCAATAAAATCAATAAGTTACAATAGGTCTTTTTTTGATTGG GTAAAATGCCAAAATCCATGATAAACACTTGTTGTAACTTATATTTTTACTATAATTTTATA
