# Supplementary material for: Analysis of CRISPR/Cas Genetic Structure, Spacer Content and Molecular Epidemiology in Brazilian Acinetobacter baumannii Clinical Isolates
Source: Pathogens. 2023 May 26;12(6):764. doi: 10.3390/pathogens12060764 (PMC10302819; doi:10.3390/pathogens12060764)
Supplement: Supplementary file 1 [file pathogens-12-00764-s001.zip › Supplementary material S3.pdf]

```
Acb_41 cas1  1   ATGGAACAAC TTAACCCATC TGACTTAAAA GCAATATTAC ATTCTAAACG AGCTAATCTG TACTACCTTG AACATTGTCG  80
AYE cas1     1   .......... .......... .......... .......... .......... .......... .......... ..........  80

Acb_41 cas1  81  AGTAATGCAA AAAGATGGGC GAGTTTTATA TCTCACTGAA GCGAAGAACG AAAACCAATA TTGGAATATT CCAATTGCTA  160
AYE cas1     81  .......... .......... .......... .......... .......... .......... .......... ..........  160

Acb_41 cas1  161 ACACCACTGT AATTTTACTT GGAACAGGTA CATCCATTAC TCAAGCTGCG ATGCGAATGT TATGTAGTGC AGGGGTATTG  240
AYE cas1     161 .......... .......... .......... .......... .......... .......... .......... ..........  240

Acb_41 cas1  241 GTGGGGTTTT GTGGAGGTGG TGGAACACCT TTATTTGCAG GTAGTGAGGT GGAGTGGTTA ACACCTCAGA GTGAATATCG  320
AYE cas1     241 .......... .......... ......G... .......... .......... .......... .......... ..........  320

Acb_41 cas1  321 ACCAACGGAA TACATGCAAG GCTGGATGAG CTTTTGGTTT GATGAAACCA AACGTTTAGA TGTTGCGAAA GCGTTTCAAT  400
AYE cas1     321 .......... .......... .......... .......... .......... .......... .......... ..........  400

Acb_41 cas1  401 TTGCCCGGAT TGAATTTATC CGCAAAATTT GGGCGAAAGA TAAAGATTTA AAAGATGAAG GTTTTTACTT AGATGACCTT  480
AYE cas1     401 .......... .......... .......... .......... .......... .......... .C..C..T.. ....A.T..G  480

Acb_41 cas1  481 GATATTCAGC AAGCCTTAAA TGGTTTTGAA AAGAAAATTC CTAACATGAC TAAGGTTGGG GATTTATTAG TTGCAGAGGC  560
AYE cas1     481 ........A. ....T..G.. .......... .......... .......... .......... ........GC ..........  560

Acb_41 cas1  561 TCAAACAACC AAACAACTTT ATAAAATTGC AGCGACTCGT TGCAAACTCA GTTTTGAACG GAACCCTGAG CAGGGTGACC  640
AYE cas1     561 .......... .......... .......... .......... .......... .......... .......... ..........  640

Acb_41 cas1  641 TTGCGAATGA CTTTTTGAAC CATGGCAATT ATTTGGCCTA TGGCCTAAGT GCGACGACAC TTTGGGTACT AGGGATTAGC  720
AYE cas1     641 .......... .......... .......... .......... .......... .......... .......... ...A......  720

Acb_41 cas1  721 CATAGTTTTG CTGTCATGCA TGGTAAAACC CGACGAGGTG CTTTGGTGTT TGATGTAGCT GATTTGATTA AAGATGCTGT  800
AYE cas1     721 .......... .G..G..... .......... .......... .......... ......G... .......... ..........  800

Acb_41 cas1  801 GGTTTTACCA TGGGCATTTA TTTGTGCTAA AGAGGGGATG AAAGAACAAG AGTTTCGTCA ACAGCTATTG CAGAAATTTA  880
AYE cas1     801 .........T .......... .......... ...A...GC. .CT..G..G. ....C..... .......... ..........  880

Acb_41 cas1  881 CAGAGTATAA ATGTCTGGAC TGGATGTTTG ATGAGGTAAA -----AGATA ---AATCTCT ACTTAAATTT AATTAG----  948
AYE cas1     881 .G..T...CG G..C..T... .......... ..C....G.. GTTAC..GC. TGT..AAG.. TTCC...... GGAG..CGAA  960

Acb_41 cas1  948 ------ 948
AYE cas1     961 TTATGA 966
```

```
Acb_41 cas3  1     ATGTTCGTAA CCTTTATTTC CCAATGCGAA AAGAAAGCGA TTCCTCGAAC TCGTCGTGTG TTAGATGCTT TTGCAGATCG  80
AYE cas3     1     ...A...... .......... ........G. .......... .......... ......C... .......... ..........  80

Acb_41 cas3  81    GATCGGTGAT AACACATGGC AAACAGTCAT TACTGAAGAT GGCTTATTGG CTGTAAAAAA ACTACTCAGA AAAACAGTCA  160
AYE cas3     81    .......... .......... .......... .......... .......... .......... .......... ..........  160

Acb_41 cas3  161   CAAAGAATAC TGCGGTGAGT TGTCATTGGA TACGGGGTCG GCGTAGAAGT GAGCTCATGT GGATAGTGGG AAATCGTAAC  240
AYE cas3     161   .......... .......... .......... .......... .......... ..A..TT.A. ..G....T.. T.....G..T  240

Acb_41 cas3  241   AAGTTTAATG AGCAGGGGAT TGTGCCTGTA AACACCACGA AAAAAAGTCT AGCTCAAAAT AAATGGGAAA ATGATTGGCA  320
AYE cas3     241   .......... .......... .......... .......... .......... .......... .......... ..........  320

Acb_41 cas3  321   TTATTTACCT CTCATTAAAG CATTGGTTGC AGTGTCTGCT TTACTTCATG ATTGGGGTAA AGCAACTGTT TTGTTTCAGC  400
AYE cas3     321   .......... .......... .......... .......... .......... .......... .......... ..........  400

Acb_41 cas3  401   AAAAACTACT GAGTAAGAAT GATCAATTCA AAGGTGATCC TTTACGACAT GAATGGATTT CCTGCATGTT GTTAAATGCT  480
AYE cas3     401   .......... .......... .......... .......... .......... .......... .......... ..........  480

Acb_41 cas3  481   TTGGTGCAAT CATCTGGTAA TACTAAGTCT GATGAAGCAT GGTTAAAACT ATTGATGAAT CAAACTTGGG ACGAGGAACT  560
AYE cas3     481   .......... .......... .......... .......... .......... .......... .......... ..........  560

Acb_41 cas3  561   ACTCAAACAA ACGATTATAA AGAACTCAGA TCAATCGAAA GTTCTAGACC AACTTCCACC TTTTGCTCAA TTAGCTGCTT  640
AYE cas3     561   .......... ......G... .......... .......... .......... .......... .......... ....T.....  640

Acb_41 cas3  641   GGTTAATTGT TTCGCATCAT CGCCTGCCGA ATTTAAAAAC TGAAAAAGAG TATAAAAAAT ATGGGAGTGA GGATATTTCA  720
AYE cas3     641   .......... .......... .......... .......... .......... .......... .......... ...C......  720

Acb_41 cas3  721   TGTATCAAGG ATTTATTTGA ATTTATCGAA GCAGATTGGG GTTATCAAAA TAAGTTTGAG GAAAAAGAGT ATCAACAGCG  800
AYE cas3     721   .......... .......... .......... .......... .......... .......... .......... ..........  800

Acb_41 cas3  801   CCTCCAATTA TGCTTCGAGT TTGAACAAGG TTTATTGACT CAATCGGTCG AATGGACCAA GCAAGTTAAA AAATGGTCAG  880
AYE cas3     801   .......... .......... .......... .......... .......C.. .......... .......... ..........  880

Acb_41 cas3  881   CGCGTTTATT ACAAGAATCT CAAGTATCAG AACAGATTTT TGTTGATGGT TGTTGGCGAG TGATTTTACA TCATGCACGC  960
AYE cas3     881   .......... .......... .......... .......... .......... .......... .......... ..........  960

Acb_41 cas3  961   TTATGTCTCA TGTTAGGAGA TCATTATTAT TCATCGTGTG AGGCTGATAA AACTTGGAAA ACAAGTTTAT CACTCGTTGC  1040
AYE cas3     961   .......... .......... .......... .......... .......... .......... .......... ..........  1040

Acb_41 cas3  1041  AAATACTGAT CCAAAAACGA AACAAGCCAA ACAGTATTTA GATGAGCATT TGGTACGTGT CAGTGACAAT GCAATGCGAG  1120
AYE cas3     1041  .......... .......... .......... .......... .......... .......... .......... ..........  1120

Acb_41 cas3  1121  TAGCCCAGTC CCTAAGCCGT TTGGCTGATG AAATGGAATC TGCATATGAT ATTCAAAAAT TAAAAAAGAA AAGTCCACAA  1200
AYE cas3     1121  ........G. .......... .......... .......... .......... .......... .......... ..........  1200

Acb_41 cas3  1201  GGTTTTGAAT GGCAAGATCA AGCTGTTAAA GGGATTCAAC AGTTCATTCA AAAAAATGAG GGGAGTGAGA AACAAGGTTG  1280
AYE cas3     1201  .......... .......... .......... ..A....... .......... .......... .......... ..........  1280

Acb_41 cas3  1281  GTTTATTGTC AACATGGCAA GTACTGGAAA AGGTAAAACC ATTGCCAATG CCAAAATTAT GCAAGCCCTA TCTCAAGATG  1360
AYE cas3     1281  .......... .......... .......... .......... .......... .......... .......... ..........  1360

Acb_41 cas3  1361  GTCAATCATT ACGTTATGTT TTAGCCTTAG GGCTAAGAAC ACTAACGTTA CAAACAGGTG ATTCATATCG TCATGATATT  1440
AYE cas3     1361  .......... .......... .......... .......... .......... .......... .......... ..........  1440

Acb_41 cas3  1441  GGTTTAAGTA GTGATGAGCT GGCGGTTTTA ATTGGTTCAA AAGCTGTACA GGAGTTACAT CATCAGGATA TAAAAAATAA  1520
AYE cas3     1441  .......... .......... .......... .......... .......... .......... .......... ..........  1520

Acb_41 cas3  1521  TCAGACAGAA GAGCTCAGCA TTGAGGAAAT AGGTTCAGAG TCATTAGAAG AACTATTAGA TAACGAACTT GATTATGATG  1600
AYE cas3     1521  .......... .......... ....A..... .......... .......... .......... .......... ..........  1600

Acb_41 cas3  1601  CTATGCCCCA AGCTGAGTTT ATGAATGCAT TATTTCCAAA GAATCAAGAG CAGCGTAATA AAGCCTTTTT GTATAAACCA  1680
AYE cas3     1601  .......... .......... .......... .......... .......... .......... .......... ..........  1680

Acb_41 cas3  1681  GTTTTGACTT GCACCATTGA TCATCTGATG GCAGCTACAG AAACCAAACG TGGCGGTAAA TATATTTTGC CAAGTTTAAG  1760
AYE cas3     1681  .......... .......... .......... .......... .......... .......... .......... ..........  1760

Acb_41 cas3  1761  GCTTTCCTCA TCTGATTTAG TCATTGATGA AGTTGATGAT TTTAATGGTC AAGACTTGAT TGCGATTGCC AGACTTATAT  1840
AYE cas3     1761  .......... .......... .T........ .......... .......... .......... .......... ..........  1840

Acb_41 cas3  1841  ATTTAGCAGG AATGTTAGGT CGGAAAGTAA TGATTTCTTC TGCAACGATT CCACCTGCAT TAGCTGAAGG ATTTTTTAAT  1920
AYE cas3     1841  .......... .......... .......... .......... .......... .......... .......... ..........  1920

Acb_41 cas3  1921  GCTTATCAGC ACGGTTGGTC GTTATATTGT GCATTTAAGA AGCTGAAAAA TATTGATACG GTTGCAATGT GGGTAGATGA  2000
AYE cas3     1921  .......... .......... .......... .......... .......... .......... .......... ..........  2000

Acb_41 cas3  2001  GTTTAAAACC AAAACTCAAA CAATTAATTC AGGTAAATCA GAAGATTTAG TACAGCAATA TAAAAAAGCA CATGATCAAT  2080
AYE cas3     2001  .......... .......... .......... .......... .......... .......... .......... ..........  2080

Acb_41 cas3  2081  TTATTGAACT TCGAGCAGAT GCGCTAAGTA AGCAAATCGT AAAGCATAAG GCTTATATTG TAGATTGTTC TGATTTGGTG  2160
AYE cas3     2081  .......... .......... .......... .......... .......... .......... .......... ..........  2160

Acb_41 cas3  2161  ACAGAAAAAG AAGTTCGACG TTTAGATCAG AGTTTGCAGT CTCAGTATTT TGAGCGGATT AAGCAAAATG CTGAACAGTT  2240
AYE cas3     2161  .......... .......... .......... .......... .......... .......... .......... ..........  2240

Acb_41 cas3  2241  GCATTTCAAA CATCATACTA TTGATACTCA AACCGGTAAA AAAGTTTCTT TTGGTGTGGT GCGAGTAGCA AATATTCCGC  2320
AYE cas3     2241  .......... .......... .......... .......... .......... .......... .......... ..........  2320

Acb_41 cas3  2321  CATGTGTTGC ACTTACTCAA TACTTACTAA ATGCTGAATG GTCTCAAGGG ATTTCACCGC GAGTGATGGC ATATCATAGT  2400
AYE cas3     2321  .......... .......... .......... .......... .......... .......... .......... ..........  2400

Acb_41 cas3  2401  CGGCAAGTGC TTTTACTACG CAGTGAACAA GAGCGACATT TAGATCGAGT CTTAAAACGG AAAGAAAAAT TAGGAGAACA  2480
AYE cas3     2401  .......... .......... .......... .......... .......... .......... .......... ..........  2480
```

```
Acb_41 cas3  2481 AACTGCTGCA TTTTTAGATG ATGTGATTCG CCAGCATCTA GATTCGACAG ATGATGAACA TGTTATTTTT ATCTTGGTTG  2560
AYE cas3     2481 .......... .......... .......... .......... .......... .......... .......... ..........  2560

Acb_41 cas3  2561 CTACACCTGT CGAAGAGGTT GGTCGAGATC ATGATTTTGA TTGGGCCATT GTAGAGCCAT CTTCTTATCG TTCTATTATT  2640
AYE cas3     2561 .......... .......... .......... .......... .......... .......... .......... ..........  2640

Acb_41 cas3  2641 CAGTTGGCTG GTCGAGTATT ACGTCATCGA AAATTAGATC AGGATATTCA AAATCCAAAT ATTGCTTTAA TGCAATATAA  2720
AYE cas3     2641 .......... .......... .......... .......... .......... .......... .......... ..........  2720

Acb_41 cas3  2721 TTTAAAAGGG TTAAGAAAGG CTAAAGTAGC ATTTGAAAAA CCTGGTTTTG AAATTAATAA TGATAAATTC AAATTGCAGA  2800
AYE cas3     2721 .......... .......... .......... .......... .......... .......... .......... ..........  2800

Acb_41 cas3  2801 CCAAAAACCT AAAAGAACTT CTTGATAACT CAGAAGCTAA TTTTAATATC AATGCTATTC CTAGAATTAA AGCAAATCAG  2880
AYE cas3     2801 .......... .......... .......... .......... .......... .......... .......... ..........  2880

Acb_41 cas3  2881 CCATTGCAAG CAATAAAAAA ATTGGCAGAC CTTGAACACG CAGTGATGGC AGATGCTTTA ACTTCATATA AGCAAGTTGG  2960
AYE cas3     2881 .......... .......... .......... .......... .......... .......... .......... ..........  2960

Acb_41 cas3  2961 AGCAAAACCA CTCAATTCAT GGTTGACACA AAAGTGGTTT TTAACTGCTT TACCTCAGAG GTTTACACCT TTTAGGCAAA  3040
AYE cas3     2961 .......... .......... .......... .......... .......... .......... .......... ..........  3040

Acb_41 cas3  3041 GCTCGCCTAA TATCCAGCTT TTTGCGGTGC CGAAAAATCA TAAGCTTGTA TTTTGTGAGA AGAATGATTT TGGTGCGTAT  3120
AYE cas3     3041 .......... .......... .......... .......... .......... .......... .......... ..........  3120

Acb_41 cas3  3121 ATTGATCGTA ATGGTTTTTA TGATATTCAT CACATTAAAT TAAGTAAGTT AGAACAAAAT CGATTATGGT TAAATCGAAA  3200
AYE cas3     3121 .......... .......... .......... .......... .......... .......... .......... ..........  3200

Acb_41 cas3  3201 TTATTACGAC ATTTTGTGTC GTTTAGCGCT TGATAAATTA GATGAAAATG AAGATGTTAA TGAAATGATA GAAGAATTAG  3280
AYE cas3     3201 .......... .......... .......... .......... .......... .......... .......... ..........  3280

Acb_41 cas3  3281 CAAAACGATA TGGGGAAATC ATGTTGCCTG AATATGATGA GGATAAACAA CTTACCTTTT CCGAGCAATT TGGACTTGTA  3360
AYE cas3     3281 .......... .......... .......... .......... .......... .......... .......... ..........  3360

Acb_41 cas3  3361 GTTTTGGATA AATAA 3375
AYE cas3     3361 .......... ..... 3375
```
