# Supplementary figures and images for: Analysis of CRISPR/Cas Genetic Structure, Spacer Content and Molecular Epidemiology in Brazilian Acinetobacter baumannii Clinical Isolates
Source: Pathogens. 2023 May 26;12(6):764. doi: 10.3390/pathogens12060764 (PMC10302819; doi:10.3390/pathogens12060764)

Tree scale: 0.1

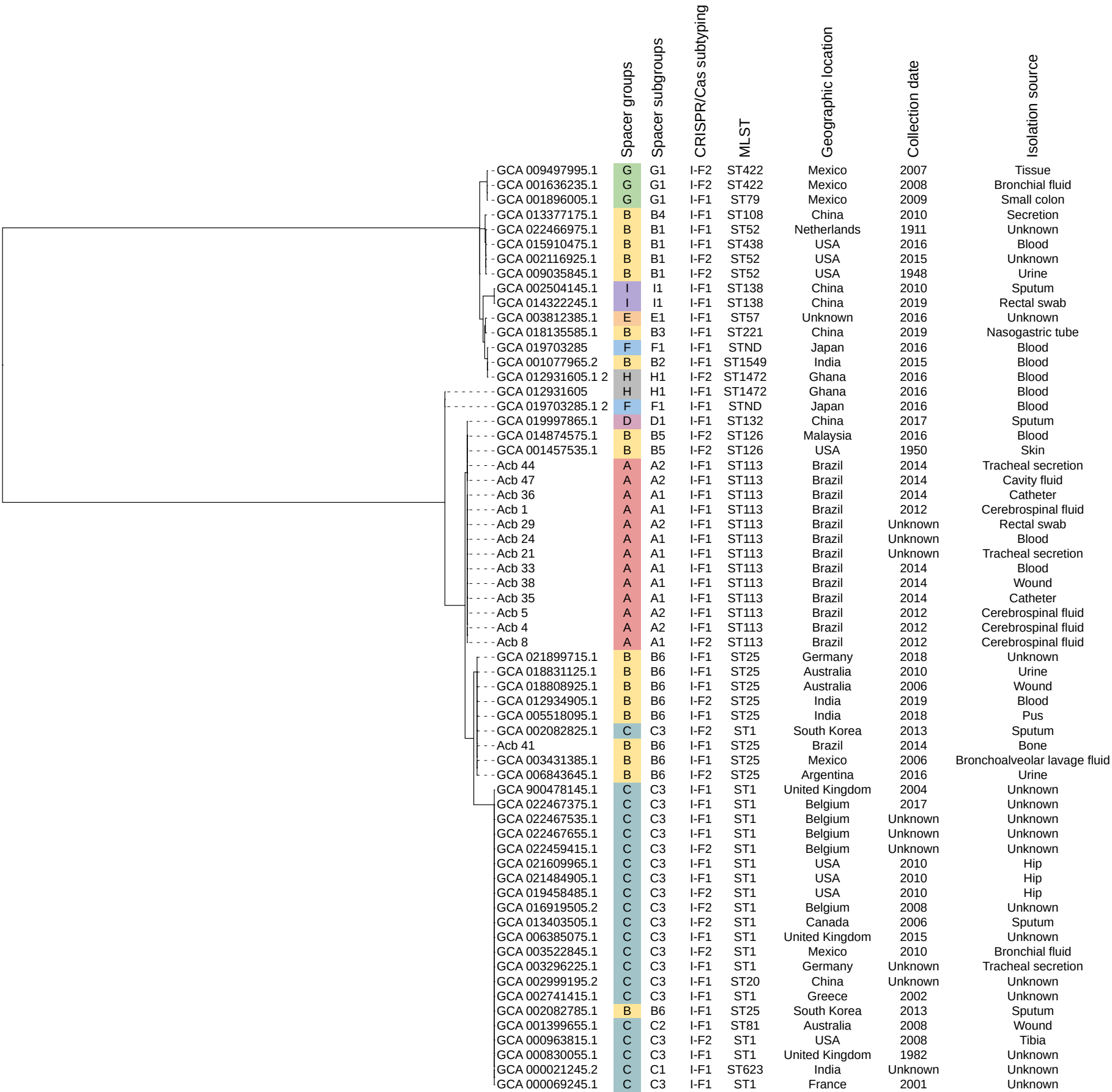

Supplement: Supplementary file 1 [file pathogens-12-00764-s001.zip › Supplementary material S8.pdf]

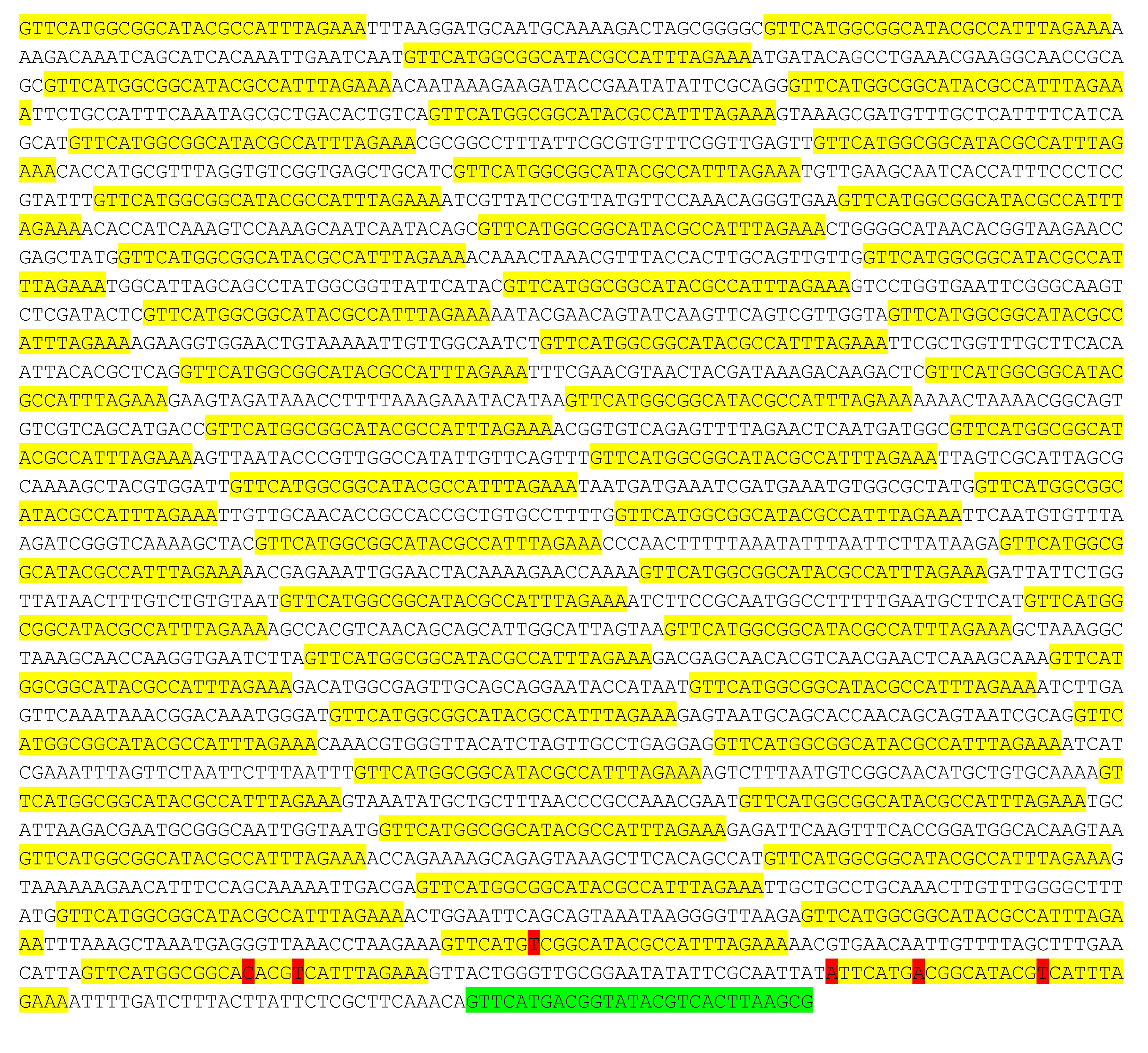

Supplement: Supplementary file 1 [file pathogens-12-00764-s001.zip › Supplementary material S1.jpg]
